# Acaricide resistance of *Rhipicephalus* (*Boophilus*)* decoloratus* (Acari: Ixodidae) on commercial farms in South Africa: filling a gap in historical data

**DOI:** 10.1007/s10493-023-00817-z

**Published:** 2023-07-06

**Authors:** Ellie M. S. P. van Dalen, Candice Jansen van Rensburg

**Affiliations:** Department of Zoology & Entomology, University of the Free State, PO Box 339, Bloemfontein, Free State South Africa

**Keywords:** Amitraz, Cypermethrin, Chlorfenvinphos, *Rhipicephalus decoloratus*, Resistance, South Africa

## Abstract

In South Africa, acaricides are widely used for tick control but very few reports are available on resistance development of *Rhipicephalus* (*Boophilus*) *decoloratus* Koch to chemical control on commercial farming systems in Africa, south of the Sahara. Resistance to different acaricide classes reported over the years was mostly from localised communal farming systems. This report addresses the lack of available information on resistance development by reviewing results found during a National Tick Resistance Survey carried out from 1998 to 2001, laying the foundation for more recent research on resistance development, and the evolution of resistance over the years. One hundred and eighty *R. decoloratus* populations were randomly collected from commercial farming systems, covering most of the provinces of South Africa. Larval immersion tests were used to determine phenotypic resistance for each tick population and 6.6% of the populations tested were found to be resistant to amitraz, 35.5% to cypermethrin, and 36.1% to chlorfenvinphos. Multi-resistance to all three acaricides was found in 1.2% of populations and a further 25.8% of the populations were resistant to two acaricides. The detection of resistance of *Rhipicephalus* (*Boophilus*) species to currently used or new acaricides is an essential tool in resistance management. The acaricides tested for the resistance of *R. decoloratus* during the survey are currently still in use in South Africa and these historical results, never published before, can be valuable and can act as reference data to determine the evolution of resistance development to acaricides in more recent studies.

## Introduction

Heavy reliance on chemical tick control for more than a century resulted in reports of resistance of *R. decoloratus* to most classes of acaricides in South Africa starting with arsenics in 1948, DDT in 1954, organophosphate and carbamate in 1966, pyrethroids in 1987, and formamidines in 1997 (George et al. 2004). In the Eastern Cape Province, Baker et al. (1978) reported resistance to organophosphate on communal areas in the eastern part, previously known as the Transkei area. In contrast, Coetzee et al. (1987) reported the total susceptibility of *R. decoloratus* to amitraz (AM) and organophosphates (OP), but high resistance to pyrethroids, including cypermethrin (CM), in a study on two field strains done at the Kwanyanga Research Station near East London. Later studies showed *Rhipicephalus* (*Boophilus*) species resistant to amidines, pyrethroids, and OP on both commercial and communal farming systems in South Africa (Mekonnen et al. 2002, 2003; Ntondini et al. 2008).

The usual practice on communal and commercial farms in the Eastern Cape and KwaZulu-Natal provinces, is to dip cattle at weekly intervals in areas close to the coast during the summer months, compared to every 2 weeks in inland areas. Winter dipping intervals were extended to 28 days at the coast, with inland dipping being suspended due to lower winter temperatures experienced (Ntondini et al. 2008). The short generation time of *R. decoloratus* can, under favourable conditions, lead to a possible completion of four generations per year (Pegram et al. 1986). Weekly dipping makes 12 acaricide exposures in 1 year possible, causing a rapid development of population resistance to the acaricide in use.

Cattle farming plays both a cultural and economic role in South Africa. Approximately 13–14 million cattle are found throughout the provinces of South Africa with a mean of 17 animals per km^2^ (DAFF 2019). A lack of information on the situation regarding the resistance of *Rhipicephalus* (*Boophilus*) species on commercial farms in South Africa, initiated a National Tick Resistance Survey (NTRS) on tick resistance from 1998 to 2001. *Rhipicephalus* (*Boophilus*) species, randomly collected from all provinces in South Africa, were tested for phenotypic resistance to AM, CM, and chlorfenvinphos (CFVP). These acaricides were mostly used for tick control during that period and are still in use in current control management strategies.

The aim of this paper was therefore to report on results obtained from commercial farms during the NTRS with the objectives being: (1) to establish the presence of acaricide resistance of *R. decoloratus* to the three classes of acaricides available for tick control in South Africa; (2) to indicate areas where populations are more prone to resistance to these acaricides; and (3) to determine the presence of multi-resistance in these areas. This information will be valuable to fill a gap in the knowledge on blue tick resistance on commercial farms in South Africa and can provide a baseline for more recent surveys to evaluate the evolution of resistant populations over the years. Although this dataset is historical in nature, control and resistance management strategies may benefit from it to plan new approaches to blue tick resistance management and to evaluate the possibility of preservation of acaricides for future use.

## Materials and methods

### Survey area and sample allocation

The nine provinces of South Africa are divided into 52 districts representing 213 local municipalities, with a total land area covering 1,220,813 km^2^ (Municipalities of South Africa 2019). Geographically, the land area consists of a wide variety of biomes and climatic zones. Eight of the nine provinces have land areas with temperate climatic conditions and cattle pastures, consisting of grasslands and wooded areas suitable for *R. decoloratus* to survive (Walker et al. 2003). From 1998 to 2001, an annual mean total number of 13,822,428 cattle (DAFF 2019), the main host for *Rhipicephalus* (*Boophilus*) species, were distributed over the eight provinces.

Stratified randomisation, using cattle densities to allocate sample sizes pro-rata to eight provinces within the distribution area of *Rhipicephalus* (*Boophilus*) species, was employed. These sample sizes were further allocated within a province by the alphabetical arrangement of specific local municipalities and sample allocations to every third or fourth municipality. Since the focus of this survey was on commercial farms, a collection of tick populations that fell partially or wholly in communal farming areas were made on farms bordering the nominated municipality.

### Sample collection and questionnaires

State veterinarians via animal health technicians and representatives of the pharmaceutical industry collected tick populations from farms in the allocated districts from 1998 to 2001. Concurrently with the collection of ticks, producers were also requested to fill out a questionnaire that included questions on acaricides used and dip frequency for the preceding 4 years. Tick collections were divided between three laboratories for resistance testing: the South African Bureau of Standards (SABS) (East London), the private laboratory of Dr. R.J. Taylor in East London, as well as the Tick Research Unit in the Department of Zoology & Entomology at the University of the Free State, Bloemfontein. Resistance profiles for each tick collection were determined by means of larval immersion tests (LIT) (Shaw 1966). A *R. decoloratus* field strain (Botshabelo), collected from cattle in a resource-poor community, served as a susceptible reference strain and was cycled on cattle on a farm close to East London. All three facilities used similar batches of commercially available compounds and tested larvae from the same reference tick collection at regular coordinated intervals to validate and standardize the procedures.

### Test procedure

Suitable females from each tick collection, identified as either *R. decoloratus* or *R. microplus*, were placed in separate Erlenmeyer flasks (ca. 20 ticks per flask) at 25 °C and > 90% relative humidity (RH). Differences in the organisation of dentition as described by Walker et al. (2003) were used to distinguish between the two tick species. After oviposition and hatching of the larvae, the larvae were exposed to the acaricides at 18–21 days after onset of the hatch, making use of the basic principles of LIT (Shaw 1966). Only four tick populations from 180 received were identified as *R. microplus*.

Three acaricide classes were tested: a commercial amidine, available as Triatix Cattle Spray (AM: 12.5% m/v, field dip concentration: 0.025% m/v, batch nr. 323); a pyrethroid available as Curatik Dip (CM: 15% m/v, field dip concentration: 0.015% m/v, batch 700,426) and an OP available as Supona 30 Cattle Dip (CFVP: 30% m/v, field dip concentration: 0.05% m/v, batch 700,109). An adapted version of LIT first described by Shaw (1966) was used. This entailed the preparation of a geometric series of seven dilutions, based on the percentage active ingredient in the commercial solution of the acaricide. Approximately 200 larvae were transferred with a soft brush to a round filter paper (Whatman no. 1, 12 cm diameter) inside a disposable aluminium plate. It was then covered with another filter paper of the same dimension, forming a larval ‘sandwich’. Ten ml of clean water used as the control, or diluted acaricide (starting with the lowest concentration) was poured onto the double layer of filter paper, which was then set aside. After 10 min, the filter paper ‘sandwich’ was opened and the two paper circles were placed on dry filter paper to absorb excessive moisture. A clean brush was used to transfer approximately 100 larvae from each circle into two replicates of dry, refolded, conical filter paper envelopes marked with the appropriate dilution. The envelopes were sealed off with a paper crimper to prevent escapees. Envelopes were stacked in sequence on racks in such a way that they did not make contact and stored at 25 °C and > 90% RH. Mortality rates were determined 72 h later, starting with the water control and lowest concentration of acaricide, and data were registered for probit analysis. Each tick population collected was exposed to all three acaricides to be able to identify populations with multi-resistant development.

### Data analysis

Data analysis were conducted at the University of the Free State. Abbott’s formula was applied to determine the corrected mortality for the various acaricide concentrations measured (Abbott 1987). The mean percentage mortality of the duplicate tests, compared to the mean of the control sample (< 10%) allowed for corrections due to incidental mortalities. The data were subjected to probit analyses using the Bio-Medical Data Package (BMDP) statistical package. This analysis included a probit transformation of % mortality and ln(x) transformation of acaricide dose. The degree of resistance was indicated by a resistance ratio (RR_50_). The RR_50_ was calculated by dividing the LC_50_ of the test population by the LC_50_ of the standard susceptible (Botshabelo) population (Ninsin 2017). Field populations with a RR_50_ of 0–49, when exposed to AM and CM, were classified as susceptible, 50–99 as emerging resistant, and > 100 as resistant populations. For CFVP a RR_50_ of > 0–2.49 was classified as susceptible, 2.5–4.9 as emerging resistant, and > 5 as resistant (Rob Taylor, pers. comm., in Mekonnen et al. 2002). Descriptive statistics applied to the RR_50_ for each acaricide, determined the mean, median, and range found for susceptible, emerging resistant and resistant populations. The factors associated with acaricide resistance at 95% confidence considered as statistically significant (α = 0.05) were analysed by making use of Microsoft Excel.

### Safety measurements

Only in vitro laboratory testing was performed and neither the ticks collected nor their progeny were at any stage introduced to their natural hosts after collection. A dedicated room used for tick resistance testing ensured both safety and prevented contamination with the concentrated remedies. The working area and biological waste bins were enclosed with double-sided tape, ensuring the capture of any stray or discarded larvae. Double-sided tape was placed around the neck of conical flasks containing the larvae. The flasks were then placed into a Petri dish filled with acetone during the assay. Brushes and forceps used for transferring larvae were placed into acetone vials directly after use, to kill the remaining larvae. Acetone treatment followed by treatment with 70% ethanol and heat treatment through submersion into boiling water, destroyed remaining adult ticks, eggs, and larvae after completing the assays. The dead ticks and larvae were filtered through a funnel lined with filter paper and were discarded into a biological waste container. A nationally registered waste disposal service periodically picked up used containers for incineration.

## Results

**Fig. 1:**
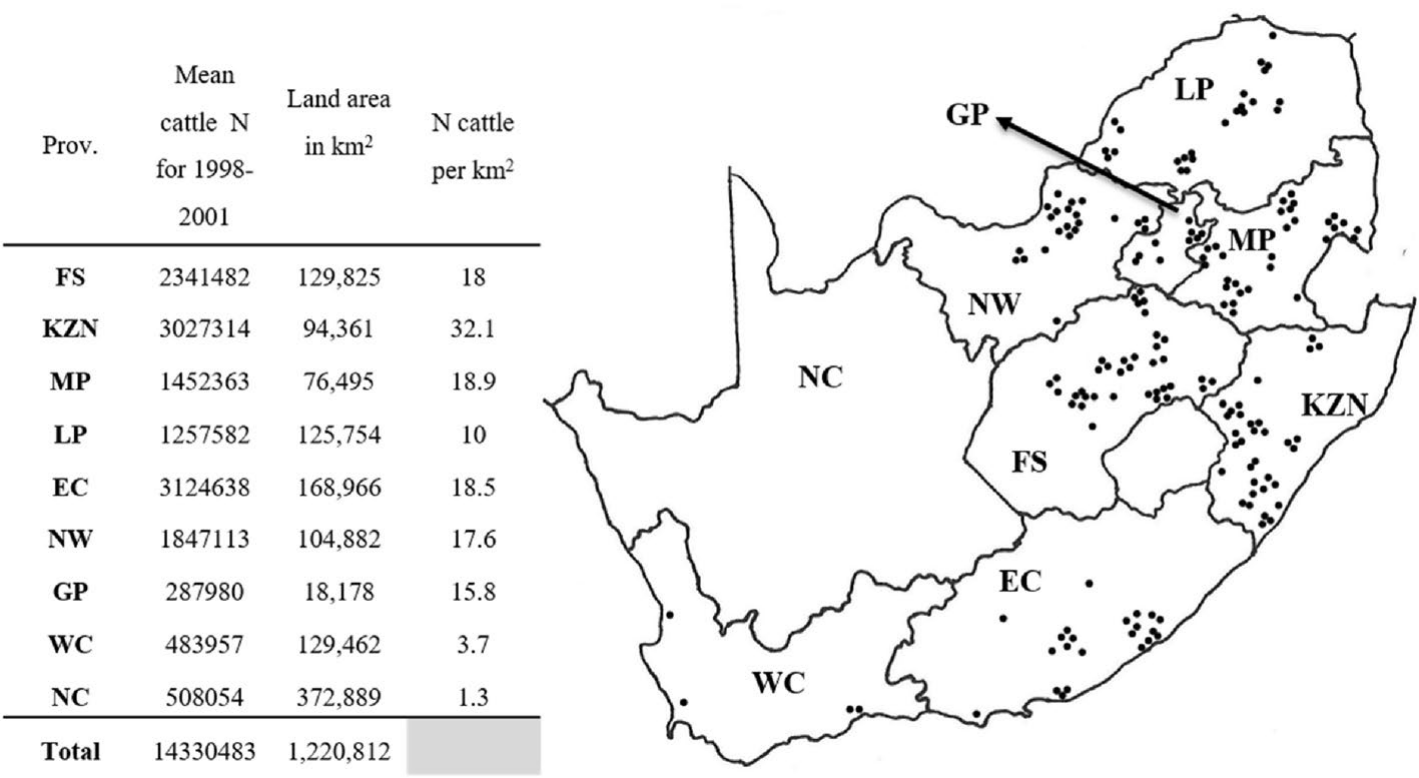
Map of collection points of field populations of *Rhipicephalus decoloratus* tested during the National Tick Resistance Survey with demographics such as mean cattle numbers for the years 1998–2001, land area (km^2^) and number of cattle per km^2^ for each province included in the embedded table. Province codes: *FS* Free State, *KZN* KwaZulu-Natal, *MP* Mpumalanga, *LP* Limpopo, *EC* Eastern Cape, *NW* North West, *GP* Gauteng, *WC* Western Cape, *NC* Northern Cape

The first usable tick sample was received during January 1998 and the last during April 2001. Two hundred and sixty-five tick populations were collected, but 85 of these collections were rejected due to ticks that were either dead on arrival, died during the incubation period, too few ticks, or the wrong tick species being collected. Eventually, 180 populations from 77 local municipal districts representing eight provinces, were tested for tick resistance (Fig. 1). Four collections identified as *R. microplus* were disregarded for evaluation in this study due to the too small sample size. Only results from tick populations identified as *R. decoloratus* were evaluated. As expected, greater numbers of tick populations were received for each province (Fig. 1) from areas having a suitable landscape, humidity as well as the number of cattle available for maintenance of tick populations (Table insert into Fig. 1). Most tick populations were obtained from the Free State Province (37), followed by KwaZulu-Natal (33), Mpumalanga (30), Limpopo (23), and the Eastern Cape and North West provinces with 22 populations each (Fig. 1). Only nine populations were obtained from Gauteng, the province with the smallest land area. Four populations were received from the Western Cape Province (Fig. 1), which also has the lowest number of cattle per km^2^ (Table insert into Fig. 1).

Populations susceptible to all three acaricides were found in 50% of populations collected and tested throughout South Africa.

### Localities of resistant populations

#### Amitraz resistance

The mean LC_50_ of the reference strain for AM as determined by the three separate laboratories was 5.3E−05. *Rhipicephalus decoloratus* populations that were either resistant or emerging resistant to AM represented 6.6% (12 populations) of the total number of 180 populations tested. Six (3.3%) populations showed resistance, with a median RR_50_ of 371.3 (100.9–9200) (Table 1). One of the six resistant populations, with a RR_50_ of 9200, was collected in the municipal district of Umvoti, located in KwaZulu-Natal Province (Tables 1, 2). One of the remaining five resistant populations was found at Piet Retief situated in Mpumalanga Province, one at Komga in the Eastern Cape Province, one at Koster in the North West Province and two in Limpopo Province, at Mokopane and Bela-Bela provincial districts (Table 2).

**Table 1:** Resistance ratio (RR_50_) and descriptive statistics for 180 *Rhipicephalus decoloratus* populations tested for resistance to amitraz, cypermethrin and chlorfenvinphos, during the National Tick Resistance Survey

	Amitraz			Cypermethrin			Chlorfenvinphos		
	S	ER	R	S	ER	R	S	ER	R
Mean	3.3	64.9	2440.0	7.3	82.2	7.4E+04	1.3	3.5	24.5
Median	0.6	62.9	371.3	1.6	82.6	823.9	1.3	3.4	8.2
Min	0.0	50.0	100.9	0.0	56.8	100.3	0.0	2.5	5.3
Max	44.7	83.2	9200.0	47.9	99.2	1.9E+06	2.5	4.9	460.4
SD	6.6	13.1	3682.1	11.3	14.8	3.0E+05	0.7	0.6	75.6
Range	44.7	33.2	9099.1	47.9	42.4	1.9E+06	2.5	2.4	455.0

The LC_50_ of the reference strain was: amitraz: 5.3E-05, cypermethrin: 3.8E-05 and chlorfenvinphos: 4.5E−04

*S* susceptible, *ER* emerging resistant, *R* resistant

Amitraz and cypermethrin: S: RR_50_ = 0–50, ER: RR_50_ = 50–99, R: RR_50_ > 100

Chlorfenvinphos: S: RR_50_ = 0–2.49, ER: 2.5–4.9, R: RR_50_ > 5

**Table 2:** The total number of field populations received from each province and district during the National Tick Resistance Survey in South Africa, indicating the number of populations susceptible (S) to all three acaricides—amitraz (AM), cypermethrin (CM), and chlorfenvinphos (CFVP)—and the number of populations that tested as resistant (R) and emerging resistant (ER) for each of the acaricides in each district

Location	No. samples	S to all three acaricides	AM 250 ppm		CM 150 ppm		CFVP 500 ppm	
			ER	R	ER	R	ER	R
Free State Province								
Bethlehem	1	1	–	–	–	–	–	–
Bothaville	3	3	–	–	–	–	–	–
Bultfontein	3	3	–	–	–	–	–	–
Edenville	1	1	–	–	–	–	–	–
Excelsior	2	2	–	–	–	–	–	–
Fouriesburg	5	4	–	–	–	–	1	–
Harrismith	3	3	–	–	–	–	–	–
Heilbron	3	2	–	–	–	–	1	–
Henneman	2	2	–	–	–	–	–	–
Kroonstad	3	2	–	–	–	1	1	–
Lindley	2	2	–	–	–	–	–	–
Parys	2	2	–	–	–	–	–	–
Ventersburg	2	2	–	–	–	–	–	–
Virginia	2	2	–	–	–	–	–	–
Vredefort	3	2	–	–	–	–	1	–
Total	37	33	0	0	0	1	4	0
KwaZulu-Natal Province								
Camperdown	1	1	–	–	–	–	–	–
Escourt	4	1	–	–	1	1	1	–
Ixopo	2	1	–	–	–	1	1	–
Klipriver	4	1	–	–	1	2	2	1
Mooi River	2	–	–	–	–	2	1	1
Msinga	1	–	–	–	–	1	–	1
Newcastle	1	–	–	–	–	1	–	1
Paulpieterburg	3	1	–	–	–	2	1	1
Pietermaritzburg	1	–	–	–	–	1	1	–
Port Shepstone	3	–	2	–	1	–	1	1
Richmond	3	–	–	–	–	3	1	2
Umvoti	3	2	–	1	–	–	–	–
Umzinto	1	1	–	–	–	–	–	–
Underburg	1	1	–	–	–	–	–	–
Weenen	3	–	–	–	2	1	1	1
Total	33	9	2	1	5	15	10	9
Mpumalanga Province								
Barberton	1		–	–	–	1	–	–
Bethal	1	1	–	–	–	–	–	–
Carolina	1	1	–	–	–	–	–	–
Delmas	2	2	–	–	–	–	–	–
Emalahleni	3	2	–	–	–	–	–	1
Ermelo	2	1	–	–	–	–	–	1
Hoëveldrif	1	–	–	–	1	–	–	–
Lydenburg	5	–	–	–	–	3	3	1
Mbombela	4	3	–	–	–	–	1	–
Nkomazi	2	–	–	–	–	2	–	1
Piet Retief	1	–	–	1	–	–	–	–
Standerton	3	2	–	–	–	–	–	1
Wakkerstroom	4	4	–	–	–	–	–	–
Total	30	16	0	1	1	6	4	5
Limpopo Province								
Bela-Bela	3	–	–	1	1	1	1	–
Lephalale	2	1	–	–	–	–	–	1
Letaba	2	1	–	–	1	–	–	–
Makhado	3	1	–	–	–	2	–	2
Modimolle	3	1	–	–	–	2	1	–
Mokopane	4	2	–	1	–	1	1	1
Musina	1	1	–	–	–	–	–	–
Polokwane	2	1	–	–	–	1	–	1
Thabazimbi	3	2	–	–	–	1	1	–
Total	23	10	0	2	2	8	4	5
Eastern Cape Province								
Adelaide	6	1	–	–	–	4	1	3
Alexandria	2	–	–	–	–	2	–	1
East London	4	–	1	–	2	2	–	3
Humansdorp	1	–	–	–	–	1	–	–
King Williams town	2	1	–	–	–	1	–	1
Komga	2	–	–	1	–	1	1	1
Pearston	1	1	–	–	–	–	–	–
Port Alfred	1	–	–	–	–	1	–	–
Queenstown	1	1	–	–	–	–	–	–
Stutterheim	2	1	–	–	–	1	–	–
Total	22	5	1	1	2	13	2	9
North West Province								
Bloemhof	1	1	–	–	–	–	–	–
Brits	3	1	–	–	–	1	1	–
Delareyville	3	3	–	–	–	–	–	–
Groot Marico	4	1	1	–	–	2	1	1
Koster	5	1	–	1	1	3	–	3
Lichtenburg	1	1	–	–	–	–	–	–
Rustenburg	1	1	–	–	–	–	–	–
Swartruggens	4	1	2	–	–	1	1	1
Total	22	10	3	1	1	7	3	5
Gauteng Province								
Alberton (1)	1	1	–	–	–	–	–	–
Bronhorstspruit	5	1	–	–	–	2	2	2
Lanseria (1)	1	1	–	–	–	–	–	–
Randfontein	2	1	–	–	–	–	–	1
Total	9	4	0	0	0	2	2	3
Western Province								
George	2	1	–	–	–	1	–	–
Malmesbury (1)	1	1	–	–	–	–	–	–
Van Rhynsdorp (1)	1	1	–	–	–	–	–	–
Total	4	3	0	0	0	1	0	0
Total for SA	180	90	6	6	11	53	29	36
% of total		50	3.3	3.3	6.1	29.4	16.1	20

A summary of populations collected for each province and the total for SA overall are also indicated

Emerging resistance to AM was detected for six of the 180 populations collected and had a median RR_50_ of 62.9 (50.0–83.2) (Table 1). These populations consisted of two from the Port Shepstone area in KwaZulu-Natal Province, one from the East London area in the Eastern Cape Province and three from the North West Province, two from the Swartruggens and one from the Groot Marico local municipalities (Table 2). No resistant or emerging resistant populations were found in the Free State, Gauteng and Western Cape provinces (Table 2).

#### Cypermethrin resistance

The mean LC_50_ of the reference strain for CM as determined by the three separate laboratories was 3.8E−05. *Rhipicephalus decoloratus* populations, either resistant or emerging resistant to CM, represented 35.5% (64 populations) of the total number of 180 populations tested during the survey. Fifty-three (29.4%) populations showed resistance, with a median RR_50_ of 823.9 (100.3–1.9E+06) (Table 1). A further 11 (6.1%) populations were indicated as emerging resistant with a median RR_50_ of 82.6 (56.8–99.2) (Table 1). Three provinces, the Free State, Western Cape, and Gauteng provinces had no emerging resistant populations and only four resistant populations were found in these provinces (Table 2). One resistant population was found in the Free State Province near Kroonstad, one in the Western Cape Province near George and two in Gauteng Province, near the Bronkhorstspruit local municipality (Table 2).

In the Mpumalanga Province, one local municipality presented with an emerging resistant population (Hoëveldrif) and six resistant populations; Barberton with one, Lydenburg with three and Nkomazi with two (Table 2). In the North West Province one population from the local municipality Koster was found to be emerging resistant and seven were resistant populations; Koster (3), Groot Marico (2), Brits (1), and Swartruggens (1) (Table 2). Limpopo Province had two populations tested as emerging resistant, one from each of the local municipalities of Letaba and Bela-Bela and eight resistant populations were found around the local municipalities of Polokwane (1), Mokopane (1), Makhado (2), Thabazimbi (1), Bela-Bela (1), and Modimolle (2) (Table 2).

In the Eastern Cape Province, two populations, both in the East London area, were emerging resistant. Thirteen resistant populations were found on farms close to Adelaide (4), Alexandria (2), Stutterheim (1), King Williamstown (1), East London (2), Komga (1), Port Alfred (1), and Humansdorp (1) (Table 2). In KwaZulu-Natal Province, five emerging resistant and 15 resistant populations were found. Emerging resistance was found on farms in the local municipalities of Escourt (1), Klipriver (1), Port Shepstone (1), and Weenen (2). Resistant populations were found in Escourt (1), Ixopo (1), Klipriver (2), Mooi River (2), Msinga (1), Newcastle (1), Paulpietersburg (2), Pietermaritzburg (1), Richmond (3), and Weenen (1) (Table 2).

#### Chlorfenvinphos resistance

The mean LC_50_ of the reference strain for CFVP as determined by the three separate laboratories was 4.5E−04. *Rhipicephalus decoloratus* populations either resistant or emerging resistant to CFVP represented 65 (36.1%) of the 180 populations tested. Thirty-six (20%) populations showed resistance, with a median RR_50_ of 8.2 (5.3–460.4). A further 29 (16.1%) populations showed emerging resistance with a median RR_50_ of 3.4 (2.53–4.9) (Table 1).

The only four populations collected from the Western Cape Province were all susceptible to CFVP. In the Free State Province, populations collected around Fouriesburg, Heilbron, Kroonstad, and Virginia local municipalities were found to be emerging resistant to CFVP. No populations resistant to CFVP were found in the Free State Province (Table 2). In Gauteng Province, two populations from farms near Bronkhorstspruit local municipality tested as emerging resistant and an additional two from the same area, as resistant. One from the Randfontein local municipality in this province also tested as resistant (Table 2). In the North West Province three emerging resistant populations, one each from Brits, Groot Marico, and Swartruggens were found. Five populations tested as resistant; near Koster (3), Groot Marico (1), and Swartruggens (1) (Table 2). In the Mpumalanga Province, four populations were emerging resistant, three of these populations were collected from farms near Lydenburg and one from the Nelspruit local municipality. In the Limpopo Province, four populations were tested as emerging resistant and five resistant (Table 2). Emerging resistant populations were shown for farms close to Mokopane, Thabazimbi, Bela-Bela, and Modimolle local municipalities. Resistant populations were found in Lepalale (1), Polokwane (1), Mokopane (1), and Makhado (2) (Table 2).

In the Eastern Cape Province, two emerging resistant populations were found; one near Adelaide and the other on a farm in the Komga local municipality. The nine populations that tested as resistant were submitted from Adelaide (3), Alexandria (1), East London (3), King Williams town (1), and Komga (1) municipalities (Table 2). In KwaZulu-Natal Province, 10 populations tested as emerging resistant and nine as resistant to CFVP. Collections from farms near the local municipalities of Escourt, Ixopo, Mooi River, Paulpietersburg, Pietermaritzburg, Port Shepstone, Richmond, and Weenen had one population each that tested as emerging resistant and Klipriver had two emerging resistant populations (Table 2). One resistant population was detected on each of the farms near Kliprivier, Mooi River, Msinga, Newcastle, Paulpietersburg, Port Shepstone, and Weenen local municipalities, and two resistant populations were found in the Richmond area (Table 3).

**Table 3:** Provincial pattern of acaricide use by farmers during the 4 years before the start of the survey (mean ± SD% of farmers completing the survey)

Province	% of completed received	Amidines (AMI)	Synthetic pyrethroids (SP)	Organophosphates (OP)	AMI/SP	SP/OP	AMI/OP	Homebrews
Free State	62	0	64.7 ± 3.1	8.4 ± 1.0	3.3 ± 1.9	20.6 ± 2.7	0	0
KwaZulu-Natal	61	31.5 ± 4.6	29.4 ± 13.5	0	28.1 ± 5.7	6.5 ± 7.3	0	0
Mpumalanga	53	5.2 ± 2.9	59.0 ± 8.8	0	19.6 ± 4.2	7.7 ± 7.0	6.2 ± 0.6	0
Limpopo	35	10.4 ± 3.7	74.8 ± 10.5	0	10.4 ± .7	1.5 ± 3.4	0	0
Eastern Cape	45	28.7 ± 8.0	39.4 ± 11.0	0	3.6	10.3 ± 1.1	0	10.3 ± 1.1
North West	32	5.6 ± 3.2	86.1 ± 6.2	5.6 ± 3.2	0	4.2 ± 3.9	0	0

Active ingredients indicated as combinations may be contained in a single compound or farmers may alternate different compounds during a particular year

### Questionnaires

**Fig. 2:**
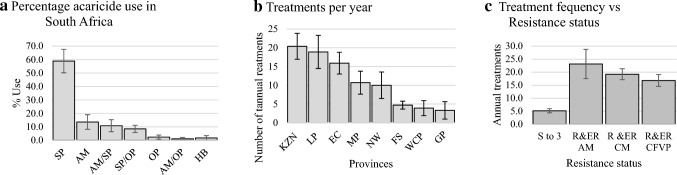
Information obtained from questionnaires indicating the mean (±SD) **a** percentage of use of each acaricide in South Africa, **b** annual number of acaricide applications made by producers for each province, and **c** relationship between annual treatments and the resistance profile found for each acaricide group. *S* susceptible, *ER* emerging resistant, *R* resistant, *SP* synthetic pyrethroids, *AM* amidines, *OP* organophosphates, *HB* homebrew mixtures, *KZN* Kwa Zulu-Natal, *LP* Limpopo, *EC* Eastern Cape, *MP* Mpumalanga Province, *FS* Free State, *WCP* Western Cape Province, *GP* Gauteng Province

Less than 50% of the 180 questionnaires handed out to producers during the survey were completed. Data reflected in the completed questionnaires were evaluated to have an indication of which acaricides were used more frequently than others (Fig. 2a) and the annual number of acaricide treatments applied to cattle herds in the various provinces (Fig. 2b).

### Acaricides used

The total use of acaricides during the 4 years preceding collection, indicated most of the producers used pyrethroids (58.9%), followed by amidines (13.6%) and a combination of an amidine and pyrethroid (10.8%) (Fig. 2a). A total of 2.3 and 1% of producers used organophosphates and a combination of amidines and organophosphates, respectively. Homebrew mixtures were used by 1.9% of the producers (Fig. 2a).

On a provincial level, most of the farmers in KwaZulu-Natal used either an amidine (31.5%), a pyrethroid (29.4%) or a combination of the two (28.1%) (Table 3). The main acaricides used in the Eastern Cape Province also consisted of a pyrethroid (39.4%) or amidine (28.7%), as well as a small percentage of both combinations of amidines/pyrethroids (3.6%) and pyrethroids/organophosphates (10.3%) (Table 3). In all the other provinces, pyrethroids were the main acaricide used; Mpumalanga Province (59%) with an amidine/pyrethroid combination of a 19.6% use, Free State Province (64.7%) with a pyrethroid/organophosphate combination of 20.6% and a low 3.3% of an amidine/pyrethroid combination. North West Province had 86% pyrethroid use (Table 3).

Too few questionnaires were received from Gauteng and the Western Cape provinces to make a meaningful evaluation.

### Frequency of treatments

The highest frequency of treatments per year was found in KwaZulu-Natal with a mean of 20.4 treatments, followed by Limpopo Province with 18.9, Eastern Cape Province with 15.9, Mpumalanga Province with 12.3, and North West Province with 10.7 treatments per year (Fig. 2b). Differences in the number of treatments between these provinces were however not significant. The only significant difference in the number of treatments was between all these provinces vs. the Free State Province (p < 0.05). The number of questionnaires received from the Western Cape and Gauteng provinces was too small to calculate significant differences.

A mean of 5.2 host treatments per year was associated with *R. decoloratus* populations susceptible to all three acaricides. This mean was significantly lower than those for populations associated with emerging resistance and resistance to AM, CM and CFVP that were treated at a mean frequency of 23.1, 19.2 and 16.8 per year, respectively (Fig. 2c).

### Multi-resistant populations

**Fig. 3:**
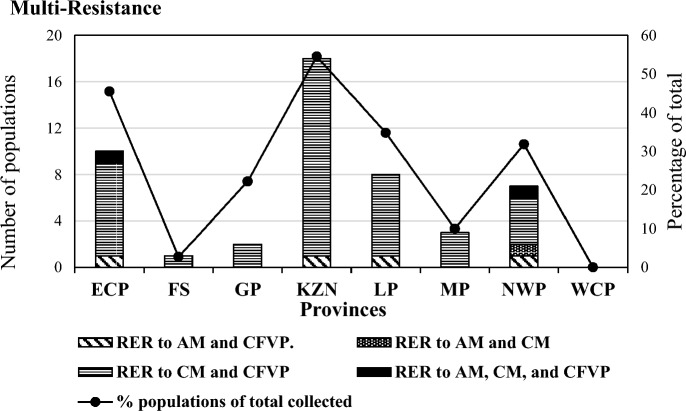
Multi-resistant populations collected for each province with each combination of acaricide-resistant populations indicated on each bar. Percentage of the total number of populations collected for each province is shown on the secondary axis (dots, line graph). *KZN* KwaZulu-Natal, *LP* Limpopo, *EC* Eastern Cape, *MP* Mpumalanga Province, *FS* Free State, *WCP* Western Cape Province, *GP* Gauteng Province, *ER* emerging resistant, *R* resistant, *AM* amitraz, *CM* cypermethrin, *CFVP* chlorfenvinphos

Populations tested nationally indicated 27.2% that were multi-resistant to two of the three acaricides, or to all three acaricides tested when populations both resistant and emerging resistant to these acaricides were evaluated. Multi-resistance to all three acaricides were found in two populations—one obtained from the Eastern Cape Province near East London that was emerging resistant to both AM and CM and resistant to CFVP, and the other one was found in the North West Province near Groot Marico that was emerging resistant to AM and resistant to both CM and CFVP. Multi-resistance to two acaricides were found in 47 of the 180 populations (25.8% of total nationally), with 42 (23.3%) multi-resistant to CM and CFVP, four (2.2%) to CFVP and AM, and one population (0.6%) to AM and CM (Fig. 3).

Populations resistant to both CM and CFVP were the main multi-resistant combination found in all provinces except the Western Cape Province. Only four populations were obtained in the Western Cape Province with no multi-resistant populations found (Fig. 3). Populations from the Free State (1; 3% of the total from province) Mpumalanga (3; 10%) and Gauteng (2; 22.1%) provinces were only multi-resistant to the combination of CM and CFVP (Fig. 3).

One population in each of the Eastern Cape, KwaZulu-Natal, Limpopo and North West Provinces was multi-resistant to AM and CFVP. The only population found to be multi-resistant to the combination of AM and CM was collected in the North West Province (Fig. 3).

On a provincial level, 54.5% of the populations collected from the KwaZulu-Natal Province were multi-resistant, followed by the Eastern Cape Province with 45.5%, Limpopo Province with 34.8%, and the North West Province with 31.8%. The other three provinces, Free State, Gauteng, and Mpumalanga, all presented with < 10% multi-resistant populations. The North West Province was the only province with multi-resistance to all acaricide combinations (Fig. 3).

## Discussion

The history of acaricide resistance in ticks plays an important role in planning tick management strategies. Although detection of resistance of *R. decoloratus* to a variety of acaricides used in South Africa throughout the years was intermittently reported, these reports were limited, and mostly restricted to communal farming areas (Baker et al. 1978; Fletcher 1984; Coetzee et al. 1987; Mekonnen et al. 2002, 2003; George et al. 2004; Ntondini et al. 2008).

Three bioassays are commonly used to test for acaricide resistance; the larval packet test (LPT) developed by Stone and Haydock (1962), the larval immersion test (LIT) first developed by Shaw (1966), and the adult immersion test (AIT), for which the protocol by Drummond et al. (1973) is most commonly used. Only the LPT and AIT were recommended for use by the FAO (1984) at the time of the NTRS. Historically the LIT, later modified by Shaw et al. (1968) by increasing post-incubation times, has been the most widely used test for tick resistance in South Africa (Baker et al. 1981; Coetzee et al. 1987). When comparing different bioassay tests to determine LC_50_ values and discriminating doses for macrocyclic lactones against the cattle tick, *R. microplus*, Sabatini et al. (2001) reported that the LIT was approximately 400 × more sensitive than the LPT. Junte (2009) also found the LIT to be more sensitive to resistance detection and suitable to detect tick resistance to most acaricides, including AM. The LIT was, therefore, the resistance test of choice for this survey.

By making use of the LIT methodology, results showed resistance of *R. decoloratus* populations to all three acaricide groups tested.

### Acaricide resistance

Phenotypic resistance to AM was found in 6.6% of the populations tested nationally. Amitraz was introduced in the early 1970s and was used worldwide to control ticks (Rodríguez-Vivas et al. 2014; Yessinon et al. 2016). Total susceptibility of *R. decoloratus* to AM was reported by Coetzee et al. (1987) in the Eastern Cape Province, with Taylor and Oberem (1995) later reporting AM resistance in South Africa in 1995. The low percentage of populations resistant to AM found during the NTRS may indicate low use of this acaricide at that time. Questionnaires confirmed that only 13% of producers made use of amidines for tick control. Two possible scenarios for AM resistance could be indicated by the results found during the NTRS. Firstly, on farms where populations resistant to only AM were found, producers might have preferred to use remedies containing only amidines without rotations or alternations with other acaricide groups. Foil et al. (2004) found that even after resistance to amidines was found, producers still preferred the use thereof on farms in Queensland. In the current study, it would seem that a higher number of treatments will be needed before resistance to AM becomes a constraint for control, representing a slow development of resistance to amidines. This could be caused by a recessive mode of inheritance, or a lower fitness found for AM-resistant ticks (Jonsson et al. 2000; Foil et al. 2004).

In the second scenario, where populations multi-resistant to AM and CM or AM and CFVP were found, the use of remedies containing AM might have become an alternative option to control ticks after the breakdown of control by remedies containing either of the other two acaricide groups. In this scenario, resistance to the previously used acaricides, either a pyrethroid or organophosphate, may still exist, whereas resistance to AM only developed after use over time. Later studies, done by Mekonnen et al. (2002, 2003) in the Eastern Cape and North West provinces, confirmed *R. decoloratus* to be resistant to AM in two of the six populations collected from commercial farming systems in South Africa. However, the sample size in these studies was too small and too soon after the NTRS to determine any significant differences. In New Caledonia, where acaricide use and tick resistance were monitored by the state, Petermann et al. (2016) found an increase of AM-resistant *R. microplus* populations from 0 to 60% in 12 years, when treated every 3–4 weeks. In South Africa, the timeframe of resistance development of *R. decoloratus* to AM is unknown and needs to be further investigated.

Cypermethrin resistance was found in 29.4%, and emerging resistance in 6.1% of the populations tested nationally. This is a high percentage for populations collected on a random basis and indicates a previous high use of pyrethroids for tick control. Coetzee et al. (1987) reported a high prevalence of resistance to pyrethroids, including CM, at the Kwanyanga Research Station near East London, which corresponded to 10 years of pyrethroid use since being introduced in 1977 (de Oliveira et al. 2012). Nolan et al. (1977) speculated that possible cross-resistance to organochlorines could have been responsible as a high resistance to DDT was previously experienced. The current results of the NTRS may be due to a combination of both high uses of pyrethroids and cross-resistance to organochlorines previously used. Results from questionnaires during the NTRS indicated that close to 60% of producers preferred pyrethroids for tick control. The most commonly used active ingredient in homebrew mixtures is also pyrethroids and although only 1.9% of producers indicated the use thereof, it is likely to be an underestimation. Those responsible for the tick collections during the NTRS also reported that producers were reluctant to admit that they used homebrew mixtures. In Mexico (state of Veracruz) and São Paulo in Brazil, 90.6 and 86% of *R. microplus* populations were resistant to pyrethroids after 15 and 20 years, respectively (Mendes et al. 2011; Fernández-Salas et al. 2012). The resistance of *R. decoloratus* to CM was also shown in two of the three populations tested by Mekonnen et al. (2002, 2003) in resistance testing done in 2000 and 2001.

Controversial reports are available for organophosphate use and resistance development in South Africa during the previous century. The first reference of resistance to organophosphates in South Africa was in the year 1966 found in a table of a publication by George et al. (2004). In a 4-year survey, Baker et al. (1978) collected 253 isolates of *R. decoloratus* in the Republic of South Africa and found 88 isolates with different degrees of resistance to organophosphate acaricides. Resistant populations to these acaricides were also reported in South Africa by Solomon et al. (1979) and Baker et al. (1981). In contrast, Coetzee et al. (1987) showed total susceptibility of *R. decoloratus* to organophosphates near East London in the Eastern Cape Province. Later, Mekonnen et al. (2002, 2003) again reported three of six *R. decoloratus* populations as resistant to CFVP in the same area. They found these results surprising as organophosphate acaricides were not used on any of the farms except for one during the previous 10 years (Mekonnen et al. 2002). During the NTRS, 16.1 and 20.0% populations tested were resistant and emerging resistant to CFVP, respectively. Questionnaires indicated that only 2.3% of the producers made use of organophosphates as tick control acaricide.

Care must, however, be taken in the interpretation of the resistance results. Although precautions were taken to survey in an unbiased manner, there is a self-selection bias inherent in resistance surveys. When high levels of tick control are achieved, one is unlikely to collect sufficient engorged ticks for resistance testing (Jonsson et al. 2000). The results of this survey provide valuable information on the relative proportions of field strains susceptible or resistant to the various groups of acaricides tested. The statistics provided on emerging resistance also alerts us to the development of potential resistance problems. These baseline data will also be useful for comparisons to more recent surveys.

### Multi-resistance

Indications of multi-resistance development to all three acaricides tested were found in two populations, one from the Eastern Cape and one from the North West Province. Multi-resistance to two acaricides was more frequently observed, with KwaZulu-Natal and the Eastern Cape being the provinces of highest concern. These two provinces presented with 54.5 and 40.8% of populations emerging resistant or resistant to two acaricides tested, respectively, with the combination of CM and CFVP multi-resistant populations the most prevalent. The high incidence of resistance to CFVP was in contrast to the low percentage use that was indicated in the questionnaires and might reflect previous usage patterns.

According to Stone (1972), ticks resistant to organophosphorus compounds maintain their resistance status in the complete absence of chemical treatment. A reversion back to susceptibility will only occur in those cases where the fitness of the resistant genotype is at a disadvantage or where the gene pool is diluted by the introduction of susceptible genes. This implies that the high use of pyrethroids during the 4 years preceding the NTRS and previous high use of organophosphates long before the NTRS may cause the high occurrence of multi-resistant populations to CM and CFVP. Furthermore, this might also be true for all the other provinces as multi-resistance to CM and CFVP were found in populations collected from all provinces, except the Western Cape Province.

Only 2.7% of multi-resistant populations were found in the Free State Province, the province with the highest number of collections tested. No populations resistant to AM were found in this province. The pattern of acaricide usage in the various provinces must, however, be viewed with caution. The statistics provided merely reflects the pattern of acaricides used by the farmers participating in the survey. It does not necessarily give an accurate picture of acaricide use for a specific province, as in many cases, sample sizes were very small. Multi-resistance shown by the NTRS may therefore have been either more or less serious than indicated by the results.

*Rhipicephalus decoloratus* collected during this survey were obtained from areas described as favourable for the promotion of tick survival due to temperate climatic conditions and alternation of grassland and wooded areas (Walker et al. 2003). These areas also satisfy the rainfall requirements for *R. decoloratus* populations, which is > 500 mm annually. Resistance development is mainly associated with high-frequency treatments, expected on farms with a high prevalence of ticks. Precipitation of > 1200 mm found along the east coast of KwaZulu-Natal and Eastern Cape provinces, together with a higher mean annual temperature in these areas (Horak et al. 2018), may be responsible for the high abundance of *R. decoloratus* throughout the year. This can cause farmers to apply treatments more often to reduce tick numbers. For example, in the Free State Province, less than five treatment applications annually are justified by lower rainfall and cold winters, which may cause tick numbers to be less abundant and more seasonal. Climate change can, however, also contribute to a new distribution range for *R. decoloratus*, and more frequent surveys need to be undertaken to keep track of its distribution and resistance development in new favourable areas.

Resistance development of *R. decoloratus* populations to acaricides in South Africa seems to follow the established pattern for *R. microplus.* Resistance development of *R. microplus* to each group of acaricide implemented for tick control, followed soon after increased use of pyrethroids (Beugnet and Chardonnet 1995; Brun 1992) and later AM (Ducornez et al. 2005). The historical review of the results obtained from the NTRS can provide a point of departure for future planning of resistance management strategies in South Africa and Africa.

This report on historic results obtained from the NTRS, and carried out on commercial farms throughout South Africa at the turn of the century, is therefore of high importance. It begins to fill a gap in the information about the extent of tick population resistance on commercial farms during the period 1998–2001 and provides baseline data for future research to evaluate the evolution of resistance throughout South Africa. It especially indicates areas more prone to tick resistance development and allows current studies on molecular detection of tick resistance to be more focused. The management program implemented on each farm within any area can have a larger contribution towards developing resistance to any acaricide in use. A greater understanding of the implications of overuse and misuse should, therefore, be strongly promoted.

### Tick control in South Africa

Compulsory dipping in South Africa was phased out after the publication of the Disease Act No. 35 of 1984, which caused the control to be the responsibility of each commercial producer. Each producer has his tick control management plan that mostly includes an unreliable history of acaricide use, with no information on the start of use up to confirmed resistance to any acaricide. The lack of knowledge on the chemical composition of acaricides, the availability of many brands on the market, as well as cost implications sometimes results in producers exacerbating problems and promoting resistance development by misuse of acaricides (Vudriko et al. 2018). Problems especially arise when rotation with alternative brands is done without realising that these brands contain the same acaricide groups as used previously; and when used in short succession results in multi-resistance. Indiscriminate increase and decrease of application concentrations and use of a homebrew mixture by producers also promote the possibility of resistance development (Vudriko et al. 2018). This results in the population resistance profile for different acaricides on each farm to be the product of a specific control management strategy, which in turn is related to acaricides used and the frequency of treatments implemented by each producer (Rodríguez-Hidalgo et al. 2017). The NTRS has also shown that if responsible management strategies are not implemented, multi-resistant tick populations will be inevitable.

## Conclusion

The historic evaluation of *R. decoloratus* resistance to acaricides fills an important information gap on resistance development of this tick species to available acaricides in South Africa. Cypermethrin was the most used acaricide during the period preceding the survey and had the highest incidence of resistant populations found in all eight provinces investigated. The level of resistance to CFVP was not significantly different from CM, although AM was indicated as the second most used acaricide. Previous high use of organophosphates and slow reversion back to susceptibility can be responsible for the high presence of resistant populations. A low percentage of populations resistant to AM was found. Multi-resistance, especially in combinations of CM and CFVP, is of great concern and was more abundantly found in the KwaZulu-Natal and Eastern Cape provinces. These two provinces are also known to have a more favourable climate for tick survival, being close to the coast and having moderate winter temperatures. It is suggested that these two provinces be monitored and managed accordingly, using more recent applications of tick control management strategies.

## Data Availability

The datasets generated during and/or analysed during the current study are available from the corresponding author on reasonable request.
